# Size-dependent antimicrobial properties of sugar-encapsulated gold nanoparticles synthesized by a green method

**DOI:** 10.1186/1556-276X-7-623

**Published:** 2012-11-12

**Authors:** Vivek D Badwaik, Lakshmisri M Vangala, Dillon S Pender, Chad B Willis, Zoraida P Aguilar, Matthew S Gonzalez, Rammohan Paripelly, Rajalingam Dakshinamurthy

**Affiliations:** 1 Department of Chemistry, Western Kentucky University, Bowling Green, KY 4210, USA; 2 Ocean Nano Tech, Springdale, AR 72764, USA

**Keywords:** Gold nanoparticles, Green synthesis, Antibacterial activity, Propidium iodide, Outer membrane vesicles

## Abstract

The antimicrobial properties of dextrose-encapsulated gold nanoparticles (dGNPs) with average diameters of 25, 60, and 120 nm (± 5) and synthesized by green chemistry principles were investigated against both Gram-negative and Gram-positive bacteria. Studies were performed involving the effect of dGNPs on the growth, morphology, and ultrastructural properties of bacteria. dGNPs were found to have significant dose-dependent antibacterial activity which was also proportional to their size. Experiments revealed the dGNPs to be bacteriostatic as well as bactericidal. The dGNPs exhibited their bactericidal action by disrupting the bacterial cell membrane which leads to the leakage of cytoplasmic content. The overall outcome of this study suggests that green-synthesized dGNPs hold promise as a potent antibacterial agent against a wide range of disease-causing bacteria by preventing and controlling possible infections or diseases.

## Background

Multidrug-resistant (MDR) bacteria represent a major threat to the success of many branches of medical sciences. Some patients are especially vulnerable of acquiring MDR bacterial infections as a consequence of treatments for illnesses such as organ transplant, hemodialysis, and various types of cancer [1]. Each year at least 150,000 people die around the world due to the infection of a particular MDR bacterium [2]. Therefore, there is an immense need for new strategies to design antibacterial agents [3].

Nanoparticles (NPs) have been used to synthesize or to improve the remedial efficacy of antibacterial agents [4-10]. NPs for this purpose are generally synthesized by using various metals and polymers [11,12]. ZnO nanoparticles have been used as an antibacterial agent but, published studies also showed that ZnO nanoparticles were toxic to T cells and neuroblastoma cells [13,14]. Copper nanoparticles have been shown as a potential antimicrobial agent, but it also comes with the cost of toxicity to eukaryotic cells e.g., potential damage to dorsal root ganglion neuron [15,16]. Similarly, silver nanoparticles have been studied extensively for their potential antibacterial properties [17], but many concerns arises over their use on mammalian cells due to the essential toxicity of silver e.g., the genotoxicity towards mammalian cell lines such as mouse embryonic stem cells and mouse embryonic fibroblasts [18,19]. The use of silver nanoparticles for medical applications is potentially limited due to their nonspecific biological toxicity. Compared to all the metal nanoparticles mentioned above, gold nanoparticles are more amenable to surface modification and are also photostable, and nontoxic based on the extensive review on nanotechnology [20,21].

Thus, where the undesirable properties such as cellular toxicity and instability of these NPs limit their application, the gold nanoparticles (GNPs) have attracted a significant interest because of their convenient surface bioconjugation, remarkable plasmon-resonant optical properties, chemical stability, and non-toxicity [22-25]. Studies have also shown that the GNPs are useful to improve the efficacy, delivery, target specificity, and biodistribution of the drugs which enhance the antibacterial activity against MDR bacteria [11-14]. However, the use of complicated non-bio/non-ecofriendly chemical synthesis processes and dependence on external sources (such as laser pulses) for the synthesis and/or the activation of GNPs limits their environmental/biocompatibility [26-30]. Moreover, the size of the GNPs strongly influences their physical, chemical, and biological properties [31,32]. Therefore, there is a need for GNPs with different sizes for various biomedical applications including those with antibacterial activity [33].

This report is focused on the antibacterial activity of the dextrose-encapsulated gold nanoparticles (dGNPs) which were synthesized by employing a ‘completely green’ method as shown in our previously published article [34]. In this context ‘green’ refers to the process in which the dGNPs were synthesized. The green process is completely natural allowing for the reproducible synthesis of differently sized dGNPs without the need for harsh chemicals or expensive equipment such as lasers. The advantages of said ‘green techniques’ are linked with natural, inexpensive, chemical stability, and ecofriendliness. Three different sizes of dGNPs were synthesized having the average diameters of 25, 60, and 120 nm (±5). The resulting dGNPs were nearly spherical, monodispersed, stable, and water soluble. The dGNPs were prepared using dextrose as both reducing and capping agent.

We explored the antibacterial activity of the dGNPs against both Gram-negative (*Escherichia coli*) and Gram-positive (*Staphylococcus epidermidis*) bacteria. Investigation of the bacterial growth kinetics and growth inhibition, in the presence of dGNPs at various concentrations, was performed using a real-time spectrophotometric assay. Antibacterial activity and efficacy were further validated by turbidimetry and spread plate assays. To understand the mechanism of action, we performed fluorescence microscopy and observed ultrathin slices of nanoparticles-treated bacterial cells under transmission electron microscope (TEM).

## Methods

Chemicals including KAuCl4/HAuCl4, Luria-Bertani broth, Luria broth (LB) agar, tryptic soy agar, propidium iodide, and dextrose were purchased from Aldrich, St. Louis, MO, USA. *E. coli* and *S. epidermidis* were purchased from Invitrogen, Carlsbad, CA, USA or obtained from Western Kentucky University’s culture collection. Analytical grade chemicals were typically used.

### Synthesis and characterization of dGNPs

Gold nanoparticles were synthesized according to our previously published environmentally benign, biofriendly, single-step/single-phase synthesis method [34]. In this method dextrose was used as a reducing agent as well as a capping agent causing the reduction of Au^3+^ ions in an aqueous buffer at room temperature and atmospheric pressure. In a typical dGNPs sample, preparation process the reaction mixture was centrifuged at 6,000 rpm for 20 min, and the supernatant was discarded. The precipitated dGNPs were resuspended in autoclaved nanopure water and then again precipitated by centrifugation (and the supernatant was discarded). This resuspension and precipitation processes were repeated at least four times and then the dGNPs samples were used for further analysis. This washing procedure helped us to make sure that the dGNPs sample does not contain any free dextrose or any free Au ions. The synthesized dGNPs were characterized using various analytical techniques including TEM analysis (Section A in Additional file 1). To determine the morphology of dGNPs, the absorption spectra were observed using Perkin Elmer Lambda 35 UV/vis spectrophotometer (Perkin Elmer Inc., Waltham, MA, USA). Energy dispersive spectroscopy was performed to analyze the elemental composition of nanoparticles using JEOL JSM 5400 LV with IXRF system (JEOL Ltd., Tokyo, Japan). Quantification of dGNPs was performed using the reported methods [3,34-38] (Section B in Additional file 1). The presence of dextrose on the surface of dGNP was further confirmed by Benedict’s test [35] (Section C in Additional file 1).The presence of hydroxyl group was determined by treatment with acetic anhydride and pyridine followed by titration with sodium hydroxide [36] (Section D Additional file 1). The more detailed experimental procedure for quantification, Benedict’s test, and volumetric titration are described in the additional word file (Additional file 1).

### Determination of antibacterial activity of dGNPs

The antibacterial experiments were performed using fresh culture of bacteria obtained by inoculating 100 μL of bacterial glycerol stock into 10 mL of sterile LB or minimal liquid medium in a culture tube. The bacteria were allowed to grow overnight in an incubator maintained at 37°C and shaken at 150 rpm. For all the experiments, dGNPs were washed several times with sterile water and then resuspended in nanopure water or medium.

### Turbidimetry

Turbidimetry refers to a microbiological assay performed for measuring the minimum inhibitory concentration (MIC) of dGNPs against *E. coli*. Various concentrations of dGNPs with average diameters of 120 nm (2 × 10^10^, 4 × 10^10^, 8 × 10^10^, 16 × 10^10^ NPs/mL), 60 nm (2 × 10^11^, 4 × 10^11^, 8 × 10^11^, 16 × 10^11^ NPs/mL), 25 nm (16 × 10^12^, 32 × 10^12^, 64 × 10^12^, 128 × 10^12^ NPs/mL) were inoculated with 1 × 10^6^ CFU/mL in a series of culture tubes containing 4 mL of sterile liquid media. Control experiments were performed by inoculating the media with *E. coli* in the absence of dGNPs. All tubes were incubated at 37°C, and the growth of bacteria was monitored by measuring the optical density (OD) at 600 nm for 12 h. OD 600 is an acronym indicating the optical density of a sample measured at a wavelength of 600 nm. Optical density at 600 nm is a common method used in molecular biology to quantify the bacteria by determining the absorbance at 600 nm [39,40].

### Spread plate technique

The bacteria (*E. coli/S. epidermidis*) were grown in a series of culture tubes containing 4 mL of sterile liquid media with various concentrations of dGNPs. Cultures from selected concentrations and growth points (12 h) were spread onto LB or tryptic soy agar plates and incubated at 37°C for 12 h to estimate the number of viable bacteria [41].

### Fluorescence assay

The bacteria were cultured in liquid media in the presence of different concentrations of dGNPs for 12 h at 37°C. The samples were collected and centrifuged at 6,000 rpm for 3 min and washed twice with phosphate buffered saline (PBS, pH ~ 7.2). These bacterial cells were incubated with propidium iodide (3 μM in PBS) in the dark for 30 min at room temperature. Fluorescence detection was performed on 10 μL of the bacterial suspension that was placed on a glass slide and observed under Leica scanning fluorescence microscope (Leica Microsystems, Buffalo Grove, IL, USA) [3]. Positive control was prepared by treating the bacterial suspension with 100% ethyl alcohol for 15 min.

### Preparation for cross section of the bacterial cells

Ultrastructural changes induced by dGNP treatment were studied under an electron microscope. The cultures of the two bacterial strains were fixed by mixing with equal volumes of a × 2 fixative solution to give final concentrations of 2.0% *w/v* paraformaldehyde and 2.5% *w/v* glutaraldehyde in 50 mM sodium cacodylate buffer (pH 7.4). After incubating with fixative for 2 h at room temperature, the fixed samples were washed and centrifuged twice; the supernatant was discarded, and the pellet was resuspended in 50 mM sodium cacodylate buffer. The same process was followed during all subsequent solution changes. Samples were post-fixed for 1 h at 25°C with 1% *w/v* osmium tetroxide in 50 mM sodium cacodylate buffer. The post-fixed samples were washed with nanopure water twice and then dehydrated in a graded ethanol series (once in 25%, 50%, 75%, and 95%, and thrice in 100% ethanol for 10 min each). The dehydrated samples were infiltrated with Spurr’s epoxy resin (once in 33%, 66%, 95%, and thrice in 100% resin for 1 h each) and then left overnight in 100% resin. The samples were centrifuged through fresh resin in BEEM capsules (BEEM Inc., West Chester, PA, USA) and hardened at 70°C for 18 h. Ultrathin sections of the pelleted samples were cut on an RMC MT-X ultra-microtome using a glass knife. The sections were stained with 2% aqueous uranyl acetate and Reynold’s lead citrate for 15 and 3 min, respectively and examined using a JEOL-100CX transmission electron microscope [42,43].

## Results and discussion

Recent advances in the field of nanobiotechnology have been used for the development of new antibacterial agents. Non-bio/ non-ecofriendly synthesis processes, cellular toxicity, and instability of nanoparticles severely limit the nanoparticles application. Taking this into consideration, we explored the antibacterial properties of stable, biocompatible, ecofriendly dGNPs.

### Synthesis and characterization of dGNPs

Three different sizes of dGNPs [25, 60, and 120 nm (±5)] were synthesized using the previously published method in which dextrose was used as a capping and reducing agent (see Section A in Additional file 1) [34]. The dGNPs of different sizes were produced by varying the concentrations of KAuCl_4_.

### Antibacterial activity

**Figure 1:**
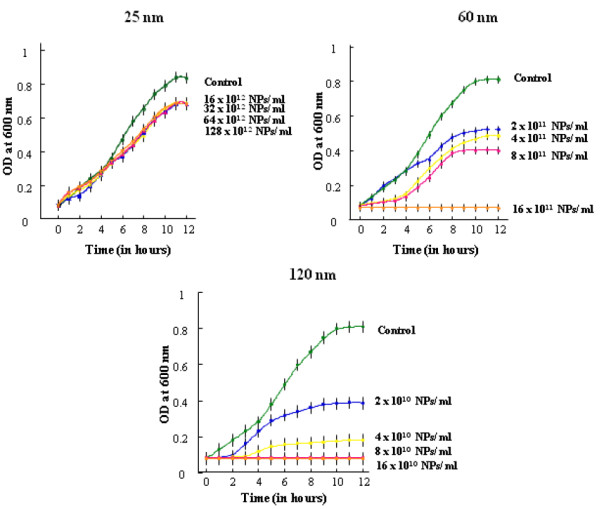
**Effect of different sizes of dextrose-encapsulated GNPs on the growth of *E. coli*.** Growth analysis curves were measured by monitoring the optical density (OD) at 600 nm, and the *E. coli* was treated with dGNPs of sizes: 25 ± 5; 60 ± 5. and 120 ± 5 nm at different concentrations (NPs/mL).

In determination of the antibacterial activity of dGNPs, we performed both liquid broth and solid agar plate-based growth studies on *E. coli*. The dGNPs of different sizes were tested for their antibacterial activity at various concentrations against *E. coli*. Before performing the actual antibacterial experiment, the dGNPs were tested for their stability by suspending the nanoparticles in sterile liquid media in culture tube and kept in an incubator shaker at 37°C. The dGNPs were found to be intact/stable (no cluster/precipitation) for more than 48 h. In the tube assay, the bacterial cells were grown in the presence of dGNPs, and the growth was monitored hourly by measuring the OD at 600 nm for 12 h. Optical density at 600 nm is a common method to quantify the concentration of bacterial cells in a liquid medium. Blank samples were prepared by suspending the dGNPs in sterile liquid medium and processed in same condition as that of the samples. Any absorption due to dGNPs was autocorrected using a blank sample. Results were plotted with the OD on *Y*-axis against the time on *X*-axis (Figure 1). Both the 120-nm and 60-nm dGNPs were found to inhibit the proliferation of *E. coli*, in a concentration-dependent manner with MIC at 16 × 10^10^ and 16 × 10^11^ NPs/mL, respectively. The 25-nm dGNPs did not significantly affect the proliferation of *E. coli* even at a concentration as high as 128 × 10^12^ NP/mL. The 120-nm dGNPs were found to be the most potent antibacterial agent compared with the 60 nm which, in turn, were more potent than the 25 nm (Figure 1). The growth kinetic data clearly suggested that the antibacterial activity was directly proportional to the increase in the size of dGNPs.

The antibacterial activity exhibited by dGNPs could be attributed to the presence of free Au^3+^ ions that remained in the solution when the dGNPs were suspended in water or due to changes in pH, since the bacterial activity is sensitive to both of these factors. To quantify the free Au^3+^ ions in the suspension, the dGNPs precipitated by centrifugation and the supernatant was tested for the presence of free Au^3+^ ions by measuring the absorbance at 290 nm. The near zero absorbance at 290 nm showed the absence of any free Au^3+^ ion (data not shown), suggesting that the free Au^3+^ ions concentration in the dGNPs suspension was insignificant and therefore, not responsible for the antibacterial activity. In addition the pH of the dGNPs suspensions was near 7.2 to 7.4 which is the normal pH range for the microorganism environment. Hence, the pH could not have been responsible for the antibacterial activity. Taken together, the results suggested that the antibacterial activity was solely due to the dGNPs and not due to free Au^3+^ ion or changes in pH.

**Figure 2:**
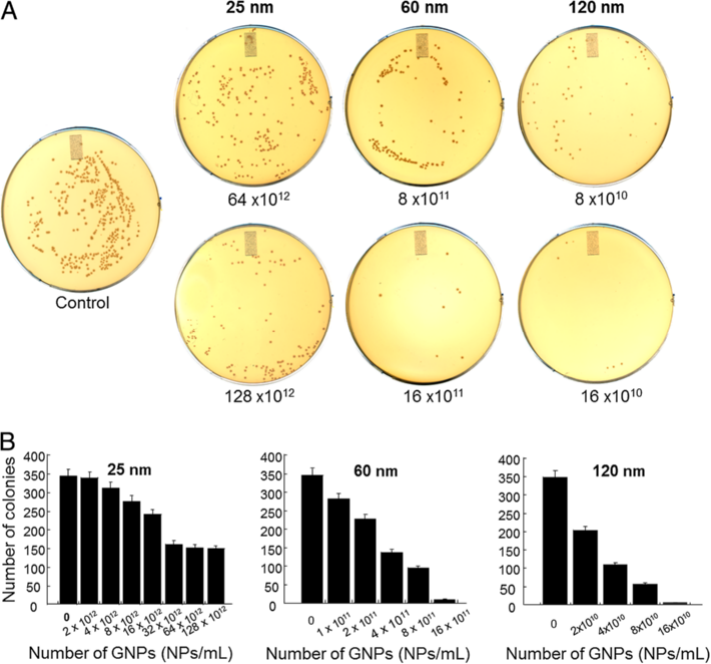
**Plate assay showing the number of viable cells recovered after treatment of *E. coli *with dGNPs.** (**A**) The bacterial cells were treated with dGNPs in the liquid media for 12 h and then were spread on the agar plates and incubated for further 12 h. (**B**) Plot of the number of *E. coli* colonies recovered against the number of dGNPs.

The results of the growth assay indicated that the presence of dGNPs inhibited the growth of *E. coli*, representing the bacteriostatic property of the dGNPs. In order to confirm whether dGNPs exhibited bactericidal action, spread plate technique was used. The cultures in the liquid broth that were treated with different sizes of dGNPs for 12 h [the time required by control to reach the stationary phase (complete growth)] were plated on fresh LB agar media. The results given in Figure 2A,B indicated that the number of colonies on the agar plate significantly decreased with increase in the size of dGNPs. The antibacterial potency of dGNPs increased as the size increased (25 < 60 < 120 nm). Furthermore, the growth of the colonies on the agar plate demonstrated that within one size of dGNPs, the bactericidal effect was concentration dependent (Figure 2). The above results from the agar spread plate methods confirmed that the dGNPs exhibited bactericidal properties.

### dGNPs-induced disruption of bacterial cells

**Figure 3:**
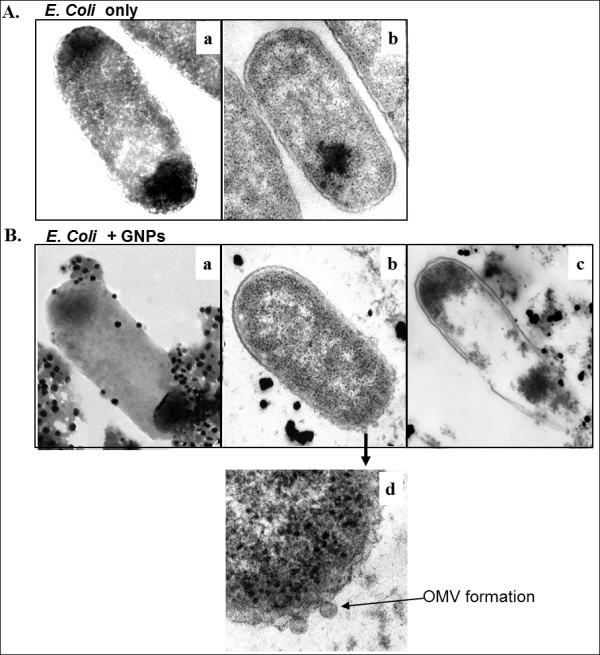
**Visualization of dGNP induced morphological changes in *E. coli *cell membranes under the TEM.** (**A**) Morphology of the untreated *E. coli* cell at 0 h (**a**); cross section of the untreated *E. coli* cell after 12 h (**b**). (**B**) Interaction of dGNPs with *E. coli* cell at 0 h (**a**); cross section of the dGNPs treated *E. coli* cell after 6 h showing the initiation of the cell disruption by the formation of outer membrane vesicles (OMVs) (**b**); cross section of the lysed *E. coli* cell after 12 h of treatment with dGNPs (**c**); the magnified view of OMV formation, which represents the initiation of disruption of cell membrane (**d**).

The process of the antibacterial activity of dGNPs was determined using time-dependent action of dGNPs on the morphology of bacterial cell and monitoring the results. This was done by collecting the dGNP-treated *E. coli* cells at different time points which were then processed for cross-sectional analysis. The TEM analysis of the cross sections of untreated cells showed that the membrane integrity remained intact at different time points even after the stationary phase (after 12 h) (a and b in Figure 3A). However, bacteria treated with 120 and 60 nm dGNPs showed gradual morphological changes within 6 h. Initially, the dGNPs were observed to anchor onto the surface of the cell at several sites (a in Figure 3B). Gradually, the sites where the dGNPs lodged showed the formation of perforations or outer membrane vesicles (OMVs) on the membrane, which eventually resulted into complete cell lysis (b and c in Figure 3B) [44,45].

**Figure 4:**
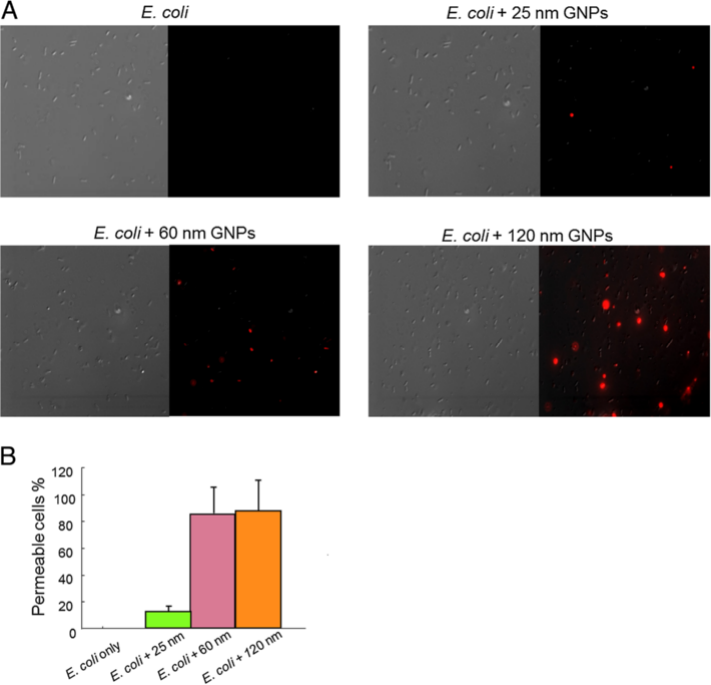
**Monitoring dGNPs-induced permeability of *E. coli *cell membranes and leakage of nucleic acids.** (**A**) The left half shows an image in the differential interference contrast mode, while the right half shows the corresponding fluorescence image. (**B**) The percentage of cells with permeable membranes from five or more fields of view obtained by two independent experiments.

To investigate the effect of various sizes of dGNPs on the cell permeability, cells were treated with propidium iodide (PI). PI is a fluorescent dye which specifically binds with nucleic acid and produces enhanced fluorescence. However, PI cannot cross intact membranes and is excluded from the viable cells [3]. Thus, in the presence of PI, cells with permeable membrane (lysed or damaged cell membrane) will fluoresce under the fluorescence microscope. For this study we incubated fresh *E. coli* cultures with different sizes of dGNPs and then treated with PI to assess the integrity of the cell membrane. Fluorescence images showed that the permeability of dGNP-treated bacteria was 92% for 120 nm, 87% for 60 nm, and very minimal permeability of less than 13% for 25 nm (Figure 4A). The permeability of the cell membrane increased significantly with exposure to increase in the size of dGNPs and also with the increase in the concentration of dGNPs (Figure 4B). Therefore, exposure of the cells to dGNPs leads to the disruption of the integrity of the cell membrane, causing increased permeability and possible leaching of the cell materials. These results indicated the size-dependent effect of the dGNPs on bacterial membrane through the time-dependent leaching/disruption. It must be noted that the mechanism of interaction between the cell membrane and the dGNPs needs to be investigated.

**Figure 5:**
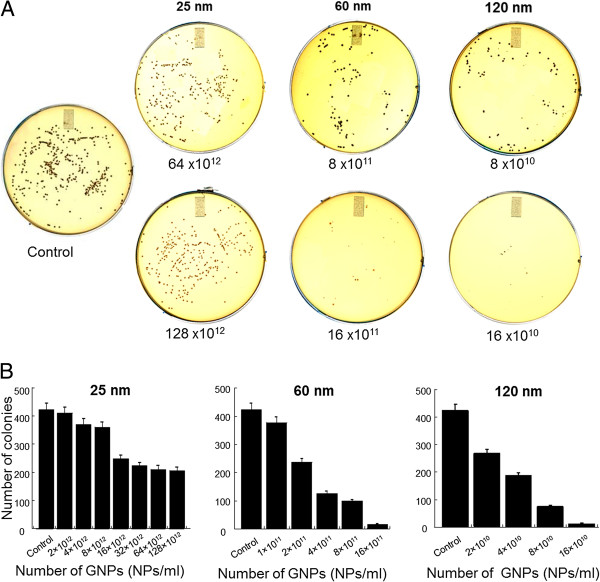
**Spread plate assays of *S. epidermidis *after treatment with dGNPs at different concentrations.** (**A**) Plate assay showing the number of viable cells recovered after the treatment of *S. epidermidis* without (control) or with dGNPs). (**B**) Graphs plotted for the number of *S. epidermidis* colonies recovered against the number of dGNPs.

**Figure 6:**
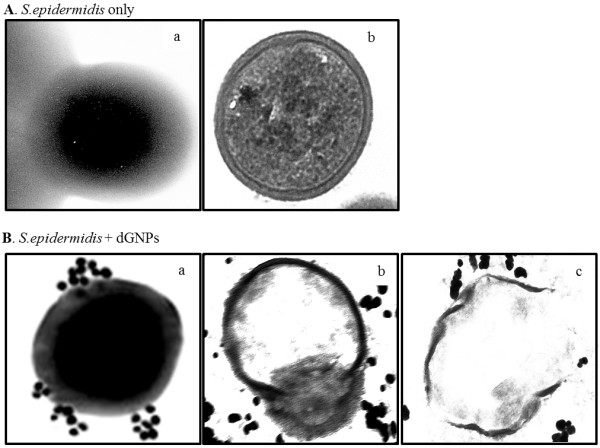
**Visualizing dGNP-induced morphological changes of *S. epidermidis *cell membranes via TEM.** (**A**) Morphology of the untreated *S. epidermidis* cells at 0 h (**a**); cross section of the untreated *S. epidermidis* cell after 12 h (**b**). (**B**) Interaction of dGNPs with *S. epidermidis* cell at 0 h (**a**); cross section of the dGNPs-treated *S. epidermidis* cell after 6 h showing the initiation of the cell wall disruption (**b**); cross section of the lysed *S. epidermidis* cell after 12 h of treatment with dGNPs (**c**).

**Figure 7:**
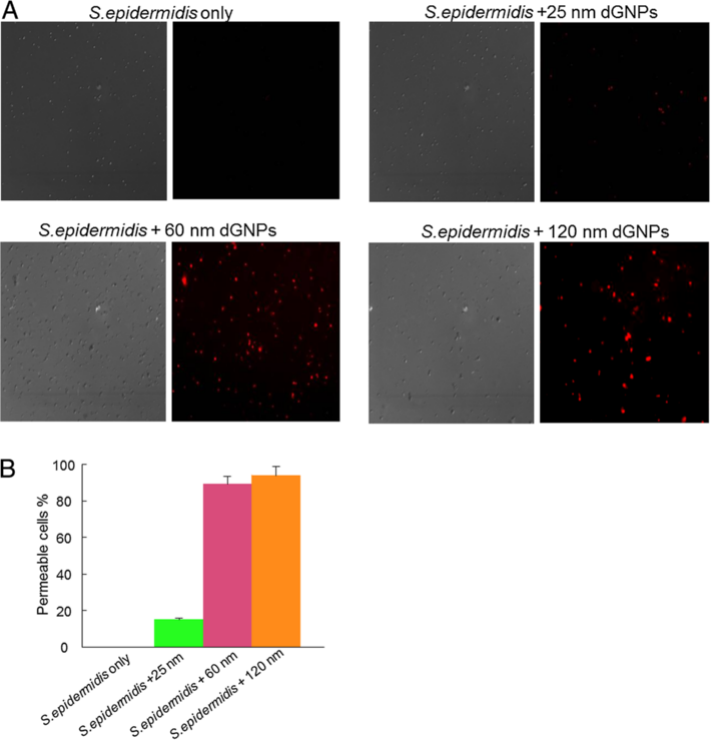
**Monitoring of dGNPs-induced permeability of *S. epidermidis *cell membranes via propidium iodide.** (**A**) For each image the left half shows an image in the differential interference contrast mode, while the right half shows the corresponding fluorescence image. (**B**) The percentage of cells with permeable membranes from five or more fields of view obtained by two independent experiments.

To determine the versatile antibacterial nature of the dGNPs, we further studied the action of dGNPs against *S. epidermidis*. *Staphylococcus* is a genus of Gram-positive bacteria, which resides normally on the skin and mucous membranes of human and other organisms causing a wide array of diseases [46-48]. Various antibacterial experiments were performed against *S. epidermidis* similar to those performed against *E. coli*. Plate-based growth studies were performed to determine the bactericidal action of dGNPs, in which the cultures in the liquid broth that were treated with different sizes of dGNPs for 12 h (complete growth) were then plated on fresh tryptic soy agar media. The results showed similarity to the results obtained against *E. coli*, indicating less number of recovered cells with increasing size of dGNPs as well as with increase in the concentration of particles (Figure 5). Though having antibiotic action against both species (*E. coli* and *S. epidermidis*), the process of action could be different. Therefore, to determine the process of action against *S. epidermidis*, TEM and fluorescence studies were performed. TEM analysis was done by collecting dGNPs-treated *S. epidermidis* cells at different time points and was processed for cross-sectional analysis. The morphological changes were observed through the analysis of these cross sections which clearly showed the disruption of cell membrane at the initial phase, which eventually lead to cell lysis (Figure 6). The fluorescence assay performed using PI also showed the disruption of the integrity of the cell membrane which causes increased permeability and possible leaching of the cell materials (Figure 7). Thus, the different types of antibacterial assays suggested that the antibacterial activity of dGNPs against *S. epidermidis* also occurred through the disruption of bacterial cellular membrane, similar to that of *E. coli*[48].

The overall results suggested similar potency and membrane disruption process for the antibacterial activity of dGNPs towards Gram-negative as well as Gram-positive bacteria. Dextrose is a polyhydroxylated molecule, which can act both as a hydrogen bond donor as well as a hydrogen bond acceptor. The presence of the capping ligand dextrose was confirmed with positive Benedict’s test (Section C in Additional file 1). The hydroxyl group was also detected using acetylation reaction followed by volumetric titration with sodium hydroxide (Section D in Additional file 1). The presence of hydroxyl group can thus be attributed for the strong electrostatic interaction between dGNPs and both Gram-negative or Gram-positive bacteria, which in turn lead to the disruption of cell membrane. Thus, this may explain the versatile antibacterial activity of these dGNPs. It may be inferred that the ligand molecule dextrose plays a crucial role in the antibacterial action but the mechanism of interaction remains to be established.

## Conclusions

In this present work, we have investigated the antibacterial activity of biofriendly/ecofriendly dextrose-encapsulated GNPs of sizes 25, 60, and 120 nm (± 5). These green-synthesized dGNPs showed significant antibacterial activity against both Gram-negative as well as Gram-positive bacteria. The efficiency of antibacterial activity was directly proportional to the increase in size as well as the concentration of dGNPs. These dGNPs were found to exert their antibacterial action via disruption of the cell membrane leading to possible leakage of the cytoplasmic contents including nucleic acids. Based on the results, it is plausible that the amphoteric nature of dextrose (the capping ligand) might be responsible for the interaction of dGNPs with both the Gram-positive and Gram-negative bacteria which, in turn, leads to the antibacterial activity. The antibacterial properties of the dGNPs hold promise for pharmaceutical, disinfectant, and other biomedical applications. The molecular level interaction between the cell membrane and the dGNPs that causes membrane rupture remains to be established.

## Competing interests

The authors declare that they have no competing interests.

## Authors’ contributions

VDB, LMV, DP, CW, and DR synthesized the different sizes of GNPs. VDB, LMV, CW, ZA, and DR characterized GNPs using TEM and performed UV/vis spectroscopy and all the antibacterial assays. All authors read and approved the final manuscript.

## Supplementary Material

Additional file 1 **Detailed description of the experimental procedures for quantification, Benedict’s test, and volumetric titration.** The additional file 1 contains the TEM images for three different sizes of dGNPs along with the description of quantification of dGNPs. The presence of dextrose on the surface of dGNPs was determined using the Benedict’s test which has been discussed along with the experimental results. Moreover, the additional file contains a brief discussion over the volumetric titration that was performed to determine the presence of unreduced hydroxyl group. Click here for file
