# Supplementary material for: Size-dependent antimicrobial properties of sugar-encapsulated gold nanoparticles synthesized by a green method
Source: Nanoscale Res Lett. 2012 Nov 12;7(1):623. doi: 10.1186/1556-276X-7-623 (PMC3533927; doi:10.1186/1556-276X-7-623)

Additional file 1

Detailed description of the experimental procedures for quantification, Benedict’s test, and volumetric titration


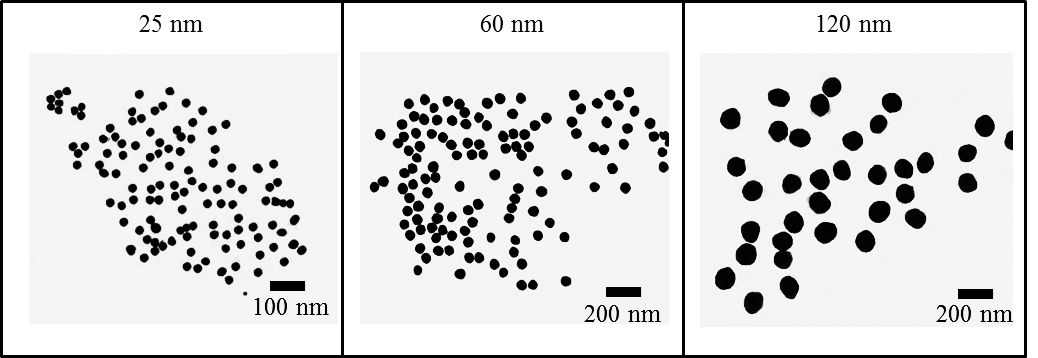


Representative TEM images of dGNPs of three sizes 25 ± 5 nm (scale bar = 100 nm); 60 ± 5 nm (scale bar = 200 nm); 120 nm (scale bar = 200 nm).

B. Quantification of dGNPs

We determine the concentration of dGNP using reported methods for calculating gold atoms per GNP (3, 27, 30 &31). One mole of any substance contains 6.023 X 10^23^ atoms (Avogadro’s number). For the synthesis of 120 nm GNP, 0.5 mM concentration of Au^3+^ was used. The total number of Au atoms in the solution = 0.500 x 10^-3^ mol/ liter x 6.023 x 10^23^ atoms/ mol= 3.012 x 10^20^ atoms/ liter i.e 3.102 x 10^17^ atoms/ mL (30, 31). We have used the reported equation, for calculating number of gold atoms, N_Au_ = (dNP/nm) ^3^ × 31, where dGNP is the diameter of spherical dGNP (3, 27). Typical dGNP of 120 nm size will have, (120nm/nm) ^3^ x 31 = 5.3568 X 10^7^ N_Au_. Therefore total number of nanoparticles (in 1 mL)/ = N_Au_ (in 1 mL sample)/ N_Au_ (in one GNP). Concentration of dGNP with average diameter of 120 nm, in one mL sample= 3.115 X 10^17^ atoms/ 5.3568 X 10^7^ atoms~ 5.622 X 10^9^ GNPs/mL. Different sizes/concentrations of dGNPs were obtained by synthesizing them in different volumes following the above described method. All values for calculation of number of dGNPs are within the error range of 500 NPs/ mL.

C. Presence of Dextrose on dGNPs

The presence of the dextrose sugar on the surface of dGNPs was determined by Benedict’s test (28). dGNPs were able to reduce copper sulphate solution which was observed by a color change from blue to brick red color. Digital images of (A) unreduced benedicts reagent and (B) reduced benedicts reagent in the presence of dGNPs are shown below.


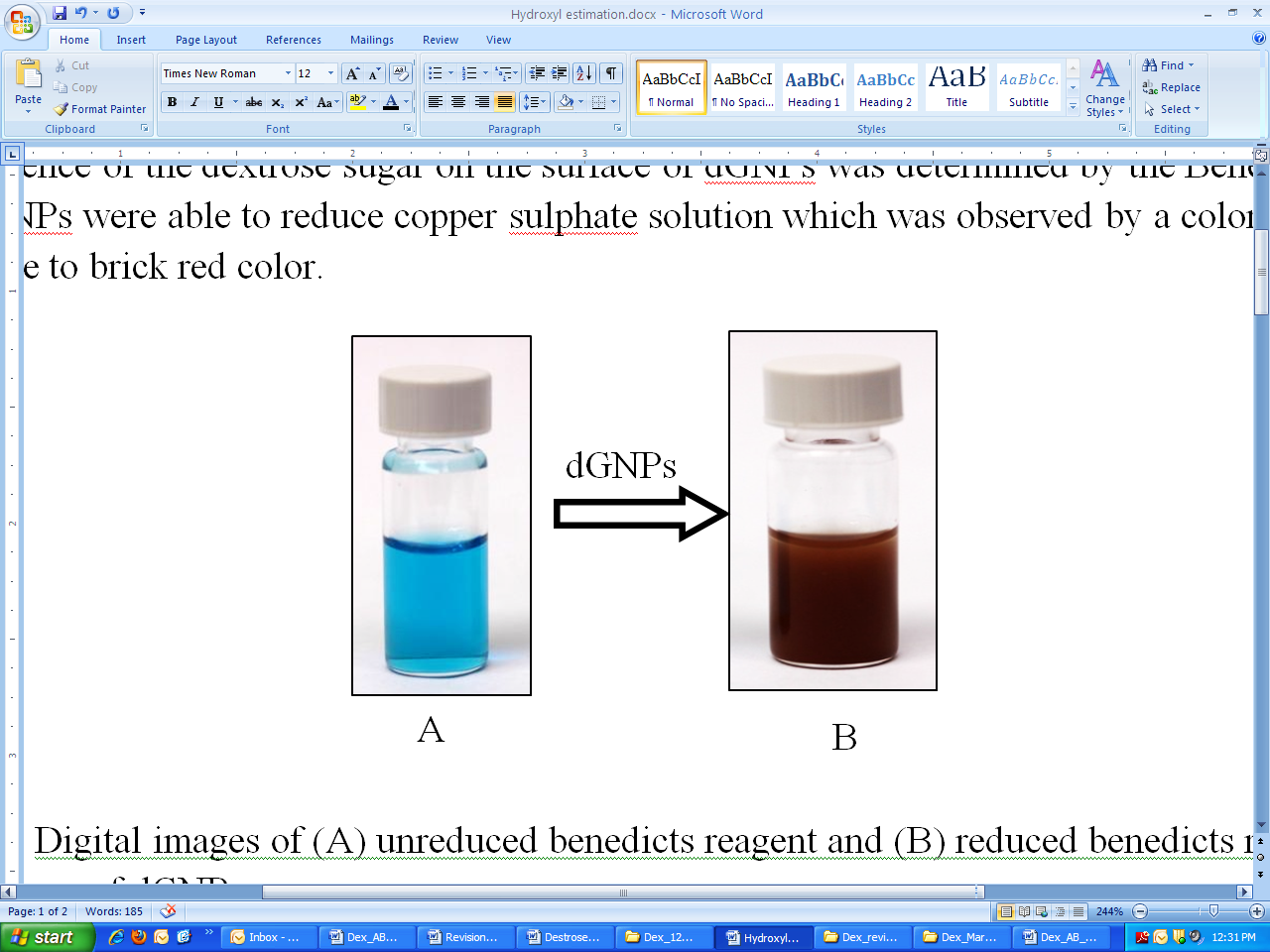


D. Presence of hydroxyl group

The presence of unreduced hydroxyl groups on the dGNPs was determined by the volumetric acid-base titration (29). In this experiment, the dGNPs were added to the mixture of pyridine and acetic anhydride (2:1). This reaction was kept for 30 hrs at 60º C for complete acetylation of hydroxyl groups. The resultant mixture was added to 200 ml ice cold water and titrated against 0.5 M sodium hydroxide with phenolphthalein indicator until the mixture turns pink. Thus the results confirmed the presence of reducing hydroxyl groups on the surface of dGNPs. Digital images of conical reaction mixture before (A) and after (B) acid-base titration indicating the end point at which the total acid (reaction mixture) in the reaction is neutralized using a base sodium hydroxide are shown below.


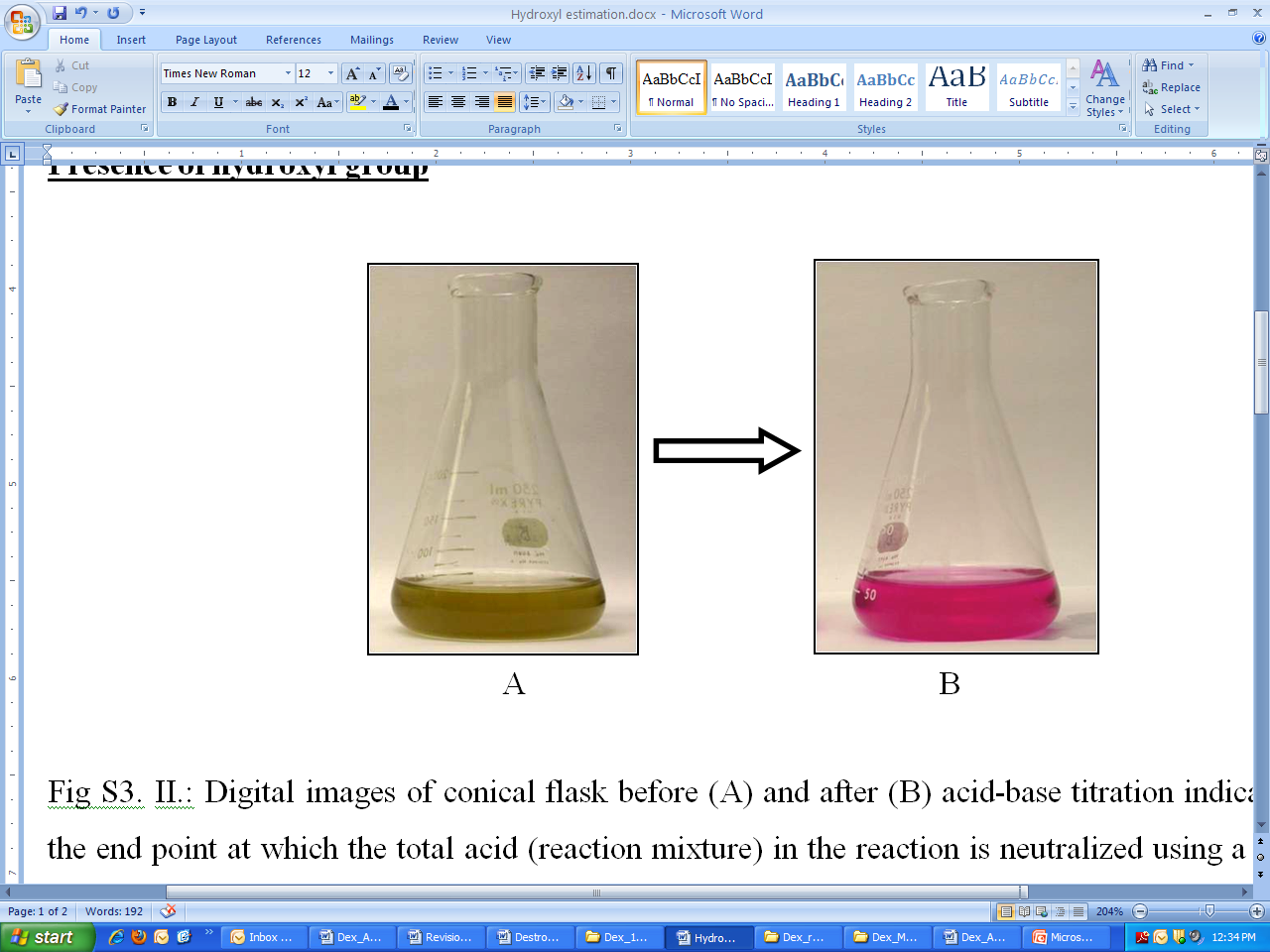

Supplement: Additional file 1 — Detailed description of the experimental procedures for quantification, Benedict’s test, and volumetric titration. The additional file 1 contains the TEM images for three different sizes of dGNPs along with the description of quantification of dGNPs. The presence of dextrose on the surface of dGNPs was determined using the Benedict’s test which has been discussed along with the experimental results. Moreover, the additional file contains a brief discussion over the volumetric titration that was performed to determine the presence of unreduced hydroxyl group. [file 1556-276X-7-623-S1.docx]
